# AI-enhanced simultaneous multiparametric ^18^F-FDG PET/MRI for accurate breast cancer diagnosis

**DOI:** 10.1007/s00259-021-05492-z

**Published:** 2021-08-10

**Authors:** V. Romeo, P. Clauser, S. Rasul, P. Kapetas, P. Gibbs, P. A. T. Baltzer, M. Hacker, R. Woitek, T. H. Helbich, K. Pinker

**Affiliations:** 1 Department of Advanced Biomedical Sciences, University of Naples Federico II, Via S. Pansini 5, 80138 Naples, Italy; 2 Department of Biomedical Imaging and Image-Guided Therapy, Division of General and Pediatric Radiology, Medical University of Vienna, Waehringer Guertel 18-20, 1090 Wien, Austria; 3 Department of Biomedical Imaging and Image-Guided Therapy, Division of Nuclear Medicine, Medical University of Vienna, Waehringer Guertel 18-20, 1090 Wien, Austria; 4 Department of Radiology, Breast Imaging Service, Memorial Sloan Kettering Cancer Center, 300 E 66th Street, New York, NY 10065 USA; 5 Department of Radiology, University of Cambridge, Cambridge, CB2 0QQ UK; 6 Department of Biomedical Imaging and Image-Guided Therapy, Division of Structural Preclinical Imaging, Medical University of Vienna, Waehringer Guertel 18-20, 1090 Wien, Austria

**Keywords:** ^18^F-FDG PET/MRI, Breast cancer, Artificial intelligence, Radiomics

## Abstract

**Purpose:**

To assess whether a radiomics and machine learning (ML) model combining quantitative parameters and radiomics features extracted from simultaneous multiparametric ^18^F-FDG PET/MRI can discriminate between benign and malignant breast lesions.

**Methods:**

A population of 102 patients with 120 breast lesions (101 malignant and 19 benign) detected on ultrasound and/or mammography was prospectively enrolled. All patients underwent hybrid ^18^F-FDG PET/MRI for diagnostic purposes. Quantitative parameters were extracted from DCE (MTT, VD, PF), DW (mean ADC of breast lesions and contralateral breast parenchyma), PET (SUVmax, SUVmean, and SUVminimum of breast lesions, as well as SUVmean of the contralateral breast parenchyma), and T2-weighted images. Radiomics features were extracted from DCE, T2-weighted, ADC, and PET images. Different diagnostic models were developed using a fine Gaussian support vector machine algorithm which explored different combinations of quantitative parameters and radiomics features to obtain the highest accuracy in discriminating between benign and malignant breast lesions using fivefold cross-validation. The performance of the best radiomics and ML model was compared with that of expert reader review using McNemar’s test.

**Results:**

Eight radiomics models were developed. The integrated model combining MTT and ADC with radiomics features extracted from PET and ADC images obtained the highest accuracy for breast cancer diagnosis (AUC 0.983), although its accuracy was not significantly higher than that of expert reader review (AUC 0.868) (*p* = 0.508).

**Conclusion:**

A radiomics and ML model combining quantitative parameters and radiomics features extracted from simultaneous multiparametric ^18^F-FDG PET/MRI images can accurately discriminate between benign and malignant breast lesions.

**Supplementary Information:**

The online version contains supplementary material available at 10.1007/s00259-021-05492-z.

## Introduction

Breast cancer is the most commonly occurring malignancy in women worldwide, representing 11.6% of newly diagnosed cancer cases in 2018 [1]. Disease prognosis changes dramatically if breast cancer is diagnosed at an early vs later stage, with the 5-year survival rate decreasing from 98 to 100% for the former to 66–98% for the latter [2]. Despite the many advantages offered by new surgical approaches and targeted drug development, early diagnosis remains one of the most effective means to conquer breast cancer.

Imaging modalities that are currently used to diagnose breast cancer include mammography, ultrasound, and magnetic resonance imaging (MRI) [3]. MRI, which is based on the depiction of neoangiogenesis as a tumor-specific feature, is the most sensitive imaging modality for breast cancer detection. However, a challenge in the broader use of breast MRI is its false-positive findings which lead to unnecessary invasive biopsies in benign tumors, along with unnecessary financial costs and patient anxiety [4]. Factors that affect MRI’s specificity include the image acquisition technique and the level of reader experience [4].

Carcinogenesis is a complex, multistep process during which cancers develop distinct pathological biological properties, i.e., cancer hallmarks, including sustained proliferation; evasion of growth suppressors and apoptosis; and promotion of angiogenesis, invasion, and metastasis [5]. Advanced imaging techniques that provide morphologic, functional, and metabolic information have been introduced, allowing the non-invasive depiction of these pathophysiological processes at the cellular level. These novel imaging data can be used for tumor diagnosis and characterization, assessment of treatment response, and prediction of patient outcome [6].

Simultaneous multiparametric ^18^F-fluoro-2-deoxy-d-glucose (^18^F-FDG) positron emission tomography/magnetic resonance imaging (PET/MRI) is a novel imaging technique that combines multiparametric morphologic and functional information from MRI with metabolic information provided by PET, offering unique insights into tumor biology to achieve the ultimate goal of precision medicine in oncology [7, 8]. Recent studies support the use of ^18^F-FDG PET/MRI in breast cancer patients for different diagnostic purposes [9, 10]. Initial studies using the combination of separately acquired MRI and PET data indicate an improvement in the discrimination of benign and malignant breast lesions [11]. However, at present, the role of simultaneous multiparametric ^18^F-FDG PET/MRI for breast cancer diagnosis has not been fully assessed.

Recently, a new paradigm in healthcare has emerged, driven by advances in medical imaging technology and image analysis as well as the advent of artificial intelligence (AI) and its applications in medical imaging. Radiomics is the extraction of large numbers of quantitative features from standard-of-care medical images using computer algorithms; radiomics features can be correlated with various variables, e.g., patient characteristics and outcomes, and pooled in large-scale analyses to create decision support models [12–14]. Radiomics has the potential to represent “the bridge between medical imaging and personalized medicine” [15].

We hypothesized that an AI-based radiomics model combining quantitative simultaneously acquired ^18^F-FDG PET/MRI data will enable an accurate discrimination of benign and malignant breast tumors. Therefore, the aim of our study was to develop and validate a diagnostic AI model using quantitative perfusion, diffusion, and metabolic data as well as radiomics features extracted from simultaneous multiparametric ^18^F-FDG PET/MRI to non-invasively discriminate between benign and malignant breast lesions.

## Materials and methods

### Patient population

**Fig. 1:**
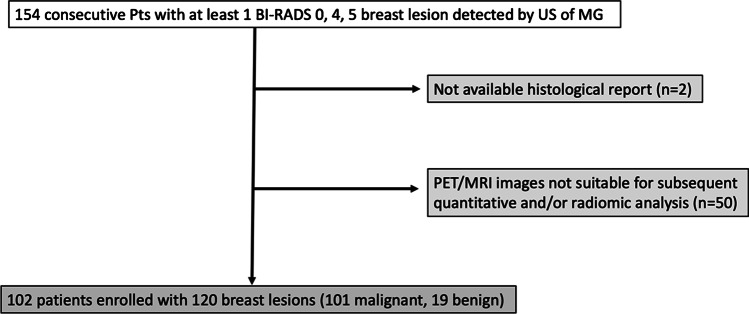
Flowchart of the patient selection process. Pts = patients; US = ultrasound; MG = mammography

This prospective single-institution study was approved by the institutional review board, and written informed consent was obtained from all participants. From June 2016 to July 2020, 154 patients were included in the study and underwent simultaneous multiparametric ^18^F-FDG PET/MRI of the breast for diagnostic purposes. Patients were included according to the following inclusion criteria: > 18 years of age; not pregnant nor breastfeeding; and imaging abnormality (i.e., Breast Imaging-Reporting and Data System (BI-RADS) 0, 4/5) on ultrasound and/or mammography (i.e., asymmetries, microcalcifications, architectural distortion, breast mass). Exclusion criteria were no histopathology or follow-up available; incomplete ^18^F-FDG PET/MRI examinations; ^18^F-FDG PET/MRI images not suitable for subsequent quantitative and radiomics analysis (e.g., image artifacts, incomplete dynamic scans); previous treatments; and contraindications for MRI examination. Thus, 102 patients (mean age 50 years, age range 23–82 years) with 120 breast lesions (101 malignant and 19 benign) were finally included in this study. The BI-RADS category distribution of included lesions was BI-RADS 0 (*n* = 8), BI-RADS 4 (*n* = 16), and BI-RADS 5 (*n* = 96). The flowchart of the patient selection process is given in Fig. 1.

### Reference standard

Histology was used as the reference standard for lesions classified as BI-RADS 4 (*n* = 22) or 5 (*n* = 95) at ^18^F-FDG PET/MRI. In patients with malignant lesions, the reference standard was histological analysis of the surgical specimen; in patients who received neoadjuvant treatment, the biopsy results were considered the reference standard. In three lesions classified as BI-RADS 2 (*n* = 2) or BI-RADS 3 (*n* = 1) at ^18^F-FDG PET/MRI, stable imaging follow-up was available for at least 2 years.

### Multiparametric PET/MRI acquisition protocol

All patients underwent simultaneous multiparametric ^18^F-FDG PET/MRI performed using a Biograph mMR system (Siemens, Germany), which is an MRI-compatible PET detector integrated with a 3.0 MRI scanner [16].

Patients fasted at least 5 h before receiving an intravenous application of ^18^F-FDG at a dose of 2.5–3.5 MBq/kg body weight. All measured blood glucose levels were less than 150 mg/dL (8.3 mmol/L) prior to tracer injection. The PET/MRI acquisition started after an uptake time of 60 min. MRI-based attenuation correction was performed using the standard Dixon-based attenuation correction method [17, 18]. A three-dimensional (3D) acquisition technique was used that offered an axial field of view (FOV) of approximately 26 cm and a transverse FOV of 59 cm with a sensitivity of 13.2 cps/kBq. Data acquisition was done for 30 min. Static PET images were reconstructed using ordinary Poisson 3D ordered subset expectation maximization (OP-OSEM) (with Gaussian scatter correction) with 3 iterations and 21 subsets into a 172-zoom 1.0 image matrix including all standard corrections (normalization, scatter, random coincidences, and decay).

Multiparametric MRI was performed using a dedicated 16-channel breast coil (Rapid Biomedical, Germany), and the imaging protocol consisted of the following sequences:

1. Axial T2-weighted sequence, repetition time/echo time (TR/TE) 4820/192 ms, matrix size 640 × 480, FOV 360 × 360 mm, slice thickness 2.5 mm, gap 3 mm, flip angle (FA) 128°.
2. Diffusion tensor imaging using a 2D diffusion-weighted (DW) single-shot spin-echo-prepared echo-planar imaging (EPI) sequence with parallel imaging and fat suppression, TR/TE 4500/87 ms, matrix size 190 × 112, FA 90°, FOV 212 × 360 mm, slice thickness 4 mm, gap 5.2 mm. Diffusion gradients were applied in twelve directions with *b* values of 0 and 800 s/mm^2^.
3. Dynamic contrast-enhanced (DCE) imaging, obtained before and after intravenous administration of a paramagnetic contrast agent (Dotarem: 0.2 ml/kg body weight), at a flow rate of 3.5 ml/s. Five pre-contrast axial T1 volumetric interpolated breath-hold examination (VIBE) sequences (FA 2°, 10°, 20°, 30°, and 40°) were first acquired for T1 mapping, followed by axial T1 Time-resolved angiography With Interleaved Stochastic Trajectories (TWIST) dynamic sequences (TR/TE 4.7/2.46 ms, matrix size 448 × 4482, FOV 340 × 340 mm, slice thickness 2 mm, no gap, flip angle 15°) with 20 measurements and a temporal resolution of 16.7 s. After an update to the clinical MRI protocol, T1 TWIST DIXON dynamic sequences were acquired (TR/TE = 4.7/1.3 ms, matrix 352 × 352, FOV 440 × 440 mm, slice thickness 2 mm, no gap, flip angle 10.5°) with 23 measurements and a temporal resolution of 14 s. Subtraction and maximum intensity projection images were then obtained.

### Image analysis

Two board-certified radiologists with 10 and 6 years of experience in breast imaging independently evaluated MRI data. A nuclear medicine physician with 10 years of experience and a radiologist with 6 years of experience who was trained in hybrid imaging under the supervision of a nuclear medicine physician independently evaluated PET images. Readers were blinded from final histopathological results and previous examinations. To assess the intraobserver reproducibility of PET/MRI quantitative parameter measurements, all lesions were reassessed by the same readers after a washout period of 4 weeks.

### Quantitative data analysis

#### Multiparametric MRI

All MR images were imported into an open-source medical image viewer (Horos v.3.3.5) for image visualization and the extraction of quantitative parameters.

Breast lesions were identified on DCE post-contrast subtracted images, and lesion location and size (maximum diameter on DCE post-contrast subtracted images in the axial plane) were recorded.

- For quantitative perfusion analysis, a pixel-by-pixel fast-deconvolution method was applied using the open-source MRI perfusion analysis tool UMMPerfusion (Horos plugin) [19]. The arterial input function was selected by drawing a 2D region of interest (ROI) in the right ventricle. Breast lesions were identified and segmented on subtracted images at early post-contrast time points, as soon as the lesions were clearly visible [20]. Two-dimensional ROIs were drawn over the enhancing tumor portion, avoiding the inclusion of cystic, hemorrhagic necrotic areas or susceptibility artifacts from biopsy markers, and then pasted onto the corresponding quantitative maps to extract the mean transit time (MTT), plasma flow (PF), and volume distribution (VD).
- DW images and corresponding quantitative apparent diffusion coefficient (ADC) maps were analyzed. First, breast lesions were identified on high *b*-value DW images; thereafter, a 2D ROI for each lesion was positioned on ADC maps on the qualitatively darkest part of the tumor, using DCE images as a reference to identify contrast-enhanced regions and also avoiding the inclusion of cystic, hemorrhagic necrotic areas or susceptibility artifacts from biopsy markers [21]. Using this approach, ADCmean of primary lesions and as well as of the normal appearing contralateral breast parenchyma was calculated.

#### ^18^F-FDG PET

**Fig. 2:**
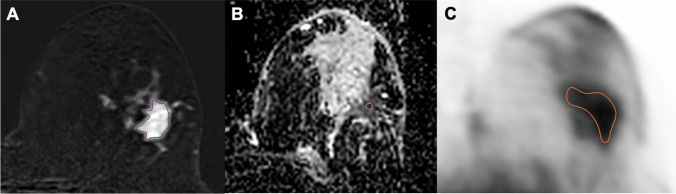
Region of interest (ROI) placement over breast lesions on dynamic contrast-enhanced magnetic resonance (DCE-MR), apparent diffusion coefficient (ADC), and positron emission tomography (PET) images for the extraction of quantitative parameters

For PET quantification, a volume of interest (VOI) was manually drawn around every suspicious breast lesion to acquire their maximum standard uptake value (SUVmax), mean SUV (SUVmean), and minimum SUV (SUVmin) using the Hermes Hybrid Viewer (Hermes Medical Solutions, Stockholm, Sweden). The VOI was defined using the region grown 3D approach with a fixed threshold to capture PET metabolic tumor volume but not physiological ^18^F-FDG uptake in the surrounding tissues. For metabolic quantification of non-tumoral ipsilateral and contralateral breast tissue, a VOI was placed in the normal breast parenchyma to obtain its SUVmean away from the nipple and areola. Examples of ROI placement over breast lesions on DCE-MRI, ADC, and PET images for the extraction of quantitative parameters are given in Fig. 2.

### Radiomics analysis and model development

**Fig. 3:**
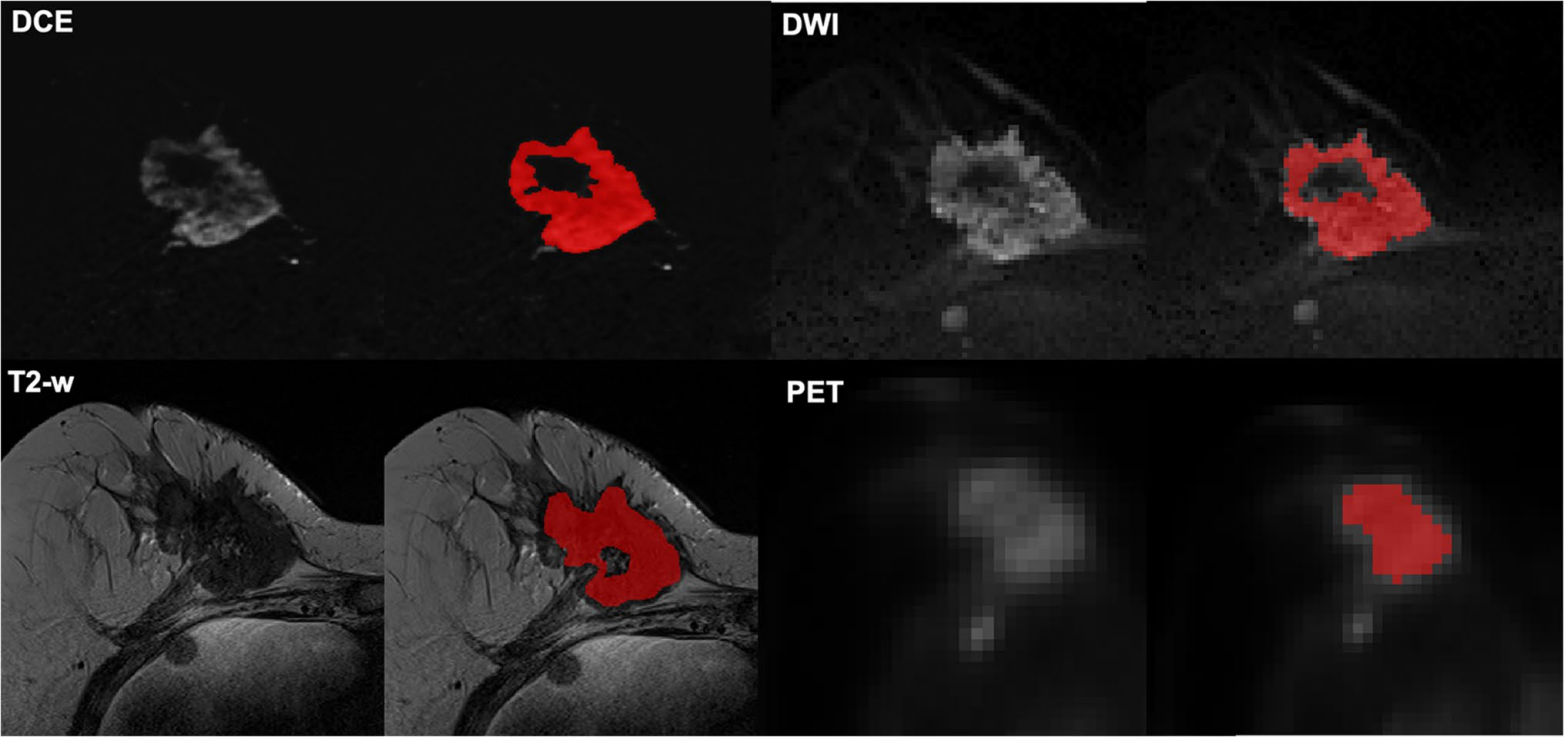
Example of tumor segmentation in a 62-year-old patient with a stage IV invasive ductal carcinoma (G3, luminal B) of the right breast. Three-dimensional regions of interest (ROIs) were drawn over breast lesions on dynamic contrast-enhanced magnetic resonance (DCE-MR) (**A**), T2-weighted (**B**), diffusion-weighted (DW) (**C**), and positron emission tomography (PET) (**D**) images using a semi-automated method

PET/MRI images were imported to dedicated software (ITK-SNAP v. 3.6.0) [22] for lesion segmentation. A radiologist with 6 years of experience in breast imaging annotated each lesion on DCE, DWI, PET, and T2-weighted images. First, whole breast lesions were segmented on DCE-MR images using a semi-automated method. The second post-contrast time point was chosen for lesion segmentation, in order to better depict tumor enhancement compared to the surrounding breast parenchyma. The same approach was applied to DWI and PET images. Finally, manual segmentation was performed to annotate breast lesions on T2-weighted images slice by slice. In all steps, care was taken to avoid the inclusion of cystic/necrotic areas. When a biopsy marker was present, a distance of at least 2 mm was kept. Examples of tumor segmentation are given in Fig. 3.

Considering the unbalanced distribution of benign and malignant breast lesions, adaptive synthetic sampling was employed to equalize class sizes [23]. Data for all four image types was initially reduced to 16 grey levels. Radiomics features were calculated using the Computational Environment for Radiological Research (CERR) [24]. DCE, T2-weighted, ADC, and PET images were used for radiomics feature extraction. Segmentations performed on DWI images were used for the extraction of radiomics features from ADC images. Considering that T2-weighted and ADC images were not isotropic, feature extraction was performed in a 2D fashion for each slice and then aggregated over the whole lesion (BTW3 as defined by the Image Biomarker Standardisation Initiative) [25]. Least Absolute Shrinkage and Selection Operator (LASSO) regression was then utilized to determine which radiomics features were of most importance. LASSO forces the sum of the regression coefficients to be less than a fixed value, which in turn forces certain coefficients to be zero, thus excluding them from affecting prediction. For this work, a maximum of the top 5 most important features were selected, to avoid overfitting the limited datasets available. LASSO was employed due to its fast nature, its ability to avoid overfitting, and the fact that it can be applied even when the number of features is greater than the number of cases/samples [26, 27]. Diagnostic models were then developed in MATLAB using a fine Gaussian support vector machine (SVM), one of the most employed machine learning (ML) classifiers in medical imaging [28]. Since there is a short supply of data to enable a split into training, validation, and test sets, the selection of ML methodology becomes especially important. An SVM was utilized since it is known to work well for small datasets, the resulting models are memory efficient (since only the coefficients corresponding to the support vectors are non-zero), they can solve both linear and non-linear problems, and they usually provide good performance [29, 30]. An SVM algorithm works by creating a hyperplane which separates the data into the desired classes. Again, since there is insufficient data to split into traditional training and test sets, fivefold cross-validation was employed since it gives the model the opportunity to train on multiple train-test splits. This results in a better indication of how well the model will perform on unseen data. Data were initially standardized (*z*-score calculation with mean 0 and standard deviation 1) to prevent dependence on any individual parameter, especially those which contain high values. This process was then repeated 1000 times to provide final diagnostic metrics. Analysis was performed for each of the four image types independently and then in various combinations to assess potential improvements in diagnostic accuracy for the discrimination of benign and malignant breast lesion.

### Clinical ^18^F-FDG PET/MRI interpretation

DCE-MRI was assessed according to the fifth edition of the BI-RADS lexicon [31]. A BI-RADS category from 2 to 5 was assigned to each lesion. BI-RADS scores were then dichotomized as follows: 2–3 = benign and 4–5 = malignant. Subsequently, ADC values were calculated for each lesion, as described above. An ADC value of 1.3 × 10^−3^ mm^2^/s was used as the diagnostic threshold for defining benignity and malignancy, as suggested by the European Society of Breast Imaging (EUSOBI) consensus statement on DW imaging [21]. Lesions showing ADC values equal to or greater than 1.3 × 10^−3^ mm^2^/s were classified as benign, while lesions with ADC values lower than 1.3 × 10^−3^ mm^2^/s were classified as malignant. On PET, a lesion was classified as benign if it did not show ^18^F-FDG uptake higher than the above background activity; conversely, a lesion showing ^18^F-FDG uptake greater than the surrounding parenchyma was classified as malignant [32]. To achieve a final diagnosis, the following criteria were applied for the combined DCE-MRI, DWI, and PET evaluation:

- A lesion was classified as malignant if at least two among DCE-MRI, DWI, and PET or all of them were positive for malignancy.
- A lesion was classified as benign if at least two among DCE-MRI, DWI, and PET or all of them were negative for malignancy.

### Statistical analysis

Intra- and interobserver reproducibility of quantitative parameter measurements was assessed using intraclass correlation coefficient (ICC) analysis. Agreement was rated as follows: poor when ICC is less than 0.5, moderate when ranging from 0.5 to 0.75, good when ranging from 0.75 to 0.90, and excellent when greater than 0.90 [33]. The Kolmogorov–Smirnov test was performed to assess whether quantitative parameters were distributed normally. The independent *t*-test or Mann–Whitney *U* test was used to compare quantitative parameters between benign and malignant breast lesions. Diagnostic accuracy, sensitivity, specificity, and positive and negative likelihood ratio of the radiologists and nuclear medicine physician’s performance in classifying breast lesions were also calculated. Receiver operating characteristic (ROC) curves of the BI-RADS score as well as significant quantitative DWI, perfusion, and PET parameters for breast cancer diagnosis were also calculated. Differences in terms of performance between the different radiomics models as well as between the best performing radiomics model and clinical interpretation were assessed using McNemar’s test. A *p* value ≤ 0.05 was considered statistically significant. Statistical analysis was performed using SPSS, version 25.0, which was released in 2017 (Armonk, NY: IBM Corp) and MedCalc 18 (MedCalc software bvba).

## Results

### Patient population

Of the 120 included breast lesions, 101 (84%) were malignant and 19 (16%) were benign. Histological features of included breast lesions are reported in Tables 1 and 2.

**Table 1:** Histological features of included malignant breast lesions

Histological diagnosis	*N*	%
Apocrine carcinoma	1	1
DCIS	3	3
IDC	79	78
ILC	7	7
IDC + ILC	3	3
Papillary carcinoma	2	2
Invasive tubular carcinoma	1	1
Lymphoma	1	1
Malignant phyllodes tumor	2	2
Mucinous carcinoma	1	1
Metaplastic carcinoma	1	1
Total	101	100
Tumor grade		
G1	7	7
G2	35	36
G3	56	57
Total	98	100
Molecular subtype		
Luminal A	10	10
Luminal B	51	52
HER2 +	12	12
Triple negative	25	26
Total	98	100

Note: *DCIS*, ductal carcinoma in situ; *HER2*, human epidermal growth factor receptor 2; *IDC*, invasive ductal carcinoma; *ILC*, invasive lobular carcinoma

**Table 2:** Final diagnosis of included benign breast lesions

Diagnosis	*N*	%	Reference standard
Sclerosing adenosis	1	5	Histology
FAH	2	11	Histology
Fibroadenoma	6	31	Histology
Fibrocystic parenchyma	1	5	Histology
Epithelial duct proliferation	2	11	Histology
Mastitis	1	5	Histology
Papilloma	2	11	Histology
PASH	1	5	Histology
BI-RADS 2 findings*	3	16	Follow-up
Total	19	100	

Note: *FAH*, fibroadenomatous hyperplasia; *PASH*, pseudoangiomatous stromal hyperplasia; *Follow-up*, clinical and instrumental follow-up. *Classified as Breast Imaging-Reporting and Data System (BI-RADS) 2 at positron emission tomography/magnetic resonance imaging (PET/MRI) and confirmed as benign during the follow-up

Breast carcinomas showed significantly higher maximum diameter (28.5 vs 10.68 mm, *p* = 0.035) and SUVmax (6.22 vs 3.09, *p* = 0.003) as well as lower ADCmean (1.42 vs 9.23 × 10^−3^ mm^2^/s, *p* < 0.001) and MTT (77.17 vs 118.90 s, *p* < 0.001) than benign breast lesions. Mean values of quantitative parameters in both benign and malignant breast lesions with corresponding significance levels are reported in Supplementary Material Table S1. ICC values of all quantitative parameters including intra- and interobserver reproducibility are shown in Supplementary Material Table S2. Intra- and interobserver reproducibility of quantitative parameter measurement ranged from moderate to excellent.

### Performance of radiomics models

A total of 101radiomic features were extracted in six classes (22 first order, 26 based on grey-level co-occurrence matrices, 16 based on run length matrices, 16 based on size zone matrices, 16 based on neighborhood grey-level dependence matrices, and five based on neighborhood grey tone difference matrices) from DCE, ADC, T2-weighted, and PET images, respectively. Eight radiomics models were developed to predict breast cancer diagnosis, based on different combinations of multiparametric ^18^F-FDG PET/MRI images. Radiomics models with corresponding selected radiomics features are reported in Table 3. Firstly, a radiomics model based on quantitative parameters alone was built. ADCmean of breast lesions, MTT, and SUVmax were selected by the LASSO regression and used by the SVM classifier, obtaining an area under the curve (AUC) of 0.981 for correctly classifying breast lesions.

**Table 3:** Selected features/quantitative parameters for each radiomics model

Radiomics model	Selected features/quantitative parameters
ADCr	ADC—minimum (FO)
	Coefficient of dispersion (FO)
	zln (SZM)
	Difference variance (GLCM)
	Inverse difference moment (GLCM)
DCE	Auto correlation (FO)
	Strength (NGTDM)
	Busyness (NGTDM)
	Standard deviation (FO)
	szlgle (SZM)
PET	Inverse difference moment normalized (GLCM)
	glv (NGLDM)
	First information correlation (GLCM)
	lzlgle (SZM)
	Skewness (FO)
T2-w	glv (SZM)
	Skewness (FO)
	glv (NGLDM)
	Kurtosis (FO)
	Correlation (GLCM)
ADCr, DCE	Minimum (FO ADC)
	Strength (NGTDM DCE)
	zp (SZM ADC)
	rln (RLM ADC)
	Coefficient of dispersion (FO ADC)
ADCr, DCE, PET	Minimum (FO ADC)
	lzlgle (SZM PET)
	Difference variance (GLCM ADC)
	Auto correlation (GLCM DCE)
	hgce (NGLDM PET)
ADCmean, MTT, SUVmax	ADCmean MTT SUVmax
ADCr, DCE, PET + ADCmean, MTT, SUVmax	lzlgle (SZM PET)
	Minimum (FO ADC)
	zln (SZM PET)
	MTT
	ADCt

Note: *ADCr*, radiomics features extracted from ADC maps; *ADCmean*, apparent diffusion coefficient mean of breast lesions; *DCE*, radiomics features extracted from dynamic contrast-enhanced images; *PET*, radiomics features extracted from positron emission tomography images; *T2-w*, radiomics features extracted from T2-weighted images; *MTT*, mean transit time of breast lesions; *SUV*, standard uptake value of breast lesions; *FO*, first-order parameter; *GLCM*, grey-level co-occurrence matrix-based parameter; *NGLDM*, neighborhood grey-level dependence matrix-based parameter; *NGTDM*, neighborhood grey tone difference matrix-based parameter; *RLM*, run length matrix-based parameter; *SZM*, size zone matrix-based parameter; *glv*, grey-level variance; *hgce*, high grey-level count emphasis; *lzlgle*, large zone low grey-level emphasis; *rln*, run length non-uniformity; *szlgle*, small zone low grey-level emphasis; *zln*, zone size non-uniformity; *zp*, zone percentage

Thereafter, the accuracy of diagnostic models based on radiomics features extracted from individual DCE, T2-weighted, ADC, and PET images was explored. Among these models, the best performance in discriminating between benign and malignant breast lesions was obtained by an SVM classifier using features extracted from ADC images (AUC 0.937, 95% confidence interval (CI): 0.901–0.973). The model based on T2-weighted features performed worse, with an AUC of 0.793 (95% CI: 0.732–0.855). Based on these findings, two radiomics models were built combining (1) radiomics features extracted from ADC maps and DCE images, and (2) radiomics features extracted from ADC, DCE, and PET images. Of these, the latter showed the best performance for breast cancer diagnosis (AUC 0.969, 95% CI: 0.947–0.990). Finally, an integrated model combining quantitative parameters and radiomics features extracted from DCE, PET images, and ADC maps was built. MTT and ADCmean of breast lesions and radiomics features extracted from ADC maps and PET images were selected by LASSO regression. This model obtained the highest accuracy in discriminating between benign and malignant breast lesions, with an AUC of 0.983 (95% CI: 0.962–1.000). A summary of all radiomics models with corresponding accuracy metrics, including area under the receiving operating characteristic curve (AUROC), diagnostic accuracy, sensitivity, specificity, and positive and negative likelihood ratio is reported in Table 4.

**Table 4:** Summary of developed radiomics models with corresponding accuracy metrics

Images	Sensitivity	Specificity	Positive likelihood ratio	Negative likelihood ratio	Accuracy	AUROC
ADCr	90.7 (83.8–93.8)	87.4 (79.4–93.1)	7.20 (4.29–11.92)	0.11 (0.05–0.19)	89.0 (84.1–93.1)	0.937 (0.901–0.973)
DCE	77.5 (67.8–85.0)	89.5 (81.7–94.6)	7.38 (4.33–14.33)	0.25 (0.18–0.36)	83.5 (77.5–88.2)	0.889 (0.841–0.937)
PET	79.4 (73.3–89.1)	83.9 (74.9–90.1)	4.93 (3.18–8.01)	0.25 (0.17–0.36)	81.7 (77.0–87.8)	0.898 (0.844–0.941)
T2-w	67.7 (57.3–76.3)	77.2 (68.4–85.3)	2.97 (2.03–4.39)	0.42 (0.31–0.57)	72.4 (65.9–78.6)	0.793 (0.732–0.855)
ADCr DCE	88.9 (81.4–94.4)	83.1 (74.9–90.1)	5.26 (3.45–8.30)	0.13 (0.07–0.23)	86.0 (80.8–90.7)	0.934 (0.901–0.968)
ADCr DCE PET	94.9 (88.8–98.4)	83.2 (74.9–90.1)	5.65 (3.69–8.82)	0.06 (0.03–0.14)	89.0 (84.1–93.1)	0.969 (0.947–0.990)
ADCmean MTT SUVmax	94.5 (87.5–97.8)	91.8 (85.3–96.6)	11.52 (6.22–23.61)	0.06 (0.02–0.13)	93.2 (88.8–96.2)	0.981 (0.966–0.996)
ADCr DCE PET + ADCmean MTT SUVmax	95.3 (88.8–98.4)	94.3 (87.6–97.8)	16.72 (7.43–35.16)	0.05 (0.02–0.12)	94.8 (90.5–97.3)	0.983 (0.962–1.000)

Note: *AUROC*, area under the receiver operating characteristic curve; *ADCr*, radiomics features extracted from ADC maps; *ADCmean*, apparent diffusion coefficient of breast lesion; *DCE*, radiomics features extracted from dynamic contrast-enhanced images; *PET*, radiomics features extracted from positron emission tomography images; *T2-w*, radiomics features extracted from T2-weighted images; *MTT*, mean transit time; *SUV*, standard uptake value. Data in brackets refer to 95% confidence intervals

Using McNemar’s test (Table 5), the performance of the integrated model combining quantitative parameters and radiomics features was higher but not significantly different from that of the other radiomics models (*p* > 0.069).

**Table 5:** Results of McNemar’s test for the comparison of area under the curve (AUC) values of the developed radiomics models

Radiomics model	ADC	DCE	PET	T2	ADCr/DCE	ADCr/DCE/PET	ADCmean/MTT/SUVmax
DCE	0.178						
PET	0.068	0.720					
T2-w	< 0.001	0.020	0.062				
ADCr/DCE	0.480	0.609	0.321	0.003			
ADCr/DCE/PET	1.000	0.178	0.068	< 0.001	0.480		
ADCmean/MTT/SUVmax	0.243	0.009	0.002	< 0.001	0.045	0.243	
ADCr DCE PET + ADCmean MTT SUVmax	0.082	0.002	< 0.001	< 0.001	0.010	0.082	0.689

Note: *DCE*, dynamic contrast enhanced; *ADC*, apparent diffusion coefficient; *PET*, positron emission tomography; *ADCr*, radiomics features extracted from ADC maps; *MTT*, mean transit time; *SUV*, standardized uptake value

### Performance of radiomics models compared to clinical ^18^F-FDG PET/MRI interpretation

The distribution of BI-RADS descriptors, ADC values, and PET findings in the study population is reported in Supplementary Material Table S3. Clinical interpretation achieved a diagnostic accuracy of 0.958 (95% CI: 0.905–0.986), sensitivity and specificity of 100% (95% CI: 96–100%) and 73.7% (95% CI: 49–91%), positive and negative likelihood ratio of 3.80 (95% CI: 1.79–8.06), and 0 in discriminating between benign and malignant breast lesions.

All radiomics models but the one based on T2-weighted radiomics features achieved greater AUCs than clinical interpretation of ^18^F-FDG PET/MRI. However, this difference was not significant (*p* = 0.508). ROC curves of BI-RADS scores along with significant quantitative DWI (ADCmean), perfusion (MTT) and PET (SUVmax) parameters for discriminating between benign and malignant breast lesions are illustrated in Supplementary Material Figure S1. Accuracy metrics of clinical interpretation, including all imaging modalities and the combined evaluation, as well as AI assessment, are summarized in Supplementary Material Table S4.

## Discussion

At present, no studies have been published on simultaneous AI-enhanced ^18^F-FDG PET/MRI for breast cancer diagnosis. The aim of this study was to investigate whether an AI-based radiomics model combining quantitative simultaneously acquired ^18^F-FDG PET/MRI data enables accurate discrimination between benign and malignant breast tumors. A model including both quantitative parameters and radiomics features was shown to accurately discriminate between benign and malignant breast lesions. Our results indicate that AI-enhanced functional and metabolic breast imaging had excellent performance and outperformed expert readers, thus having the potential to assist human readers in correctly classifying suspicious breast lesions and obviate unnecessary invasive breast procedures.

While DCE-MRI is undisputedly the most sensitive test for breast cancer detection, with a pooled sensitivity of 99% [34], there is still room for improvement in its diagnostic accuracy due to factors including overlap in imaging features between benign and malignant breast tumors, interpretation-influencing physiological factors such as background parenchymal enhancement, and last but not least human detection or interpretation error [35].

To compensate for these limitations, additional functional and metabolic imaging techniques such as DWI, perfusion imaging, and PET have been developed that provide insights into tumor biology and thus improve diagnostic accuracy. Several studies have shown the incremental diagnostic value of these individual parameters [36, 37]; particularly, their combined application as multiparametric MRI or PET/MRI has been shown to improve diagnostic accuracy for breast cancer detection and characterization [11, 38].

Our findings also indicate that different functional and metabolic imaging techniques enable the non-invasive simultaneous depiction of oncogenic processes such as induction of neoangiogenesis, metabolic reprogramming, and sustained proliferation. In our study, the clinical interpretation of ^18^F-FDG PET/MRI showed good diagnostic accuracy with an AUC of 0.868 for breast cancer diagnosis, in line with previous studies [11, 38, 39].

To fully leverage the wealth of information provided by simultaneous multiparametric ^18^F-FDG PET/MRI, we aimed to develop and validate a diagnostic AI model using quantitative perfusion, diffusion, and metabolic data as well as radiomics features to non-invasively differentiate benign from malignant breast lesions.

The AI model with the best diagnostic accuracy was based both on radiomics features extracted from ADC and PET images as well as the quantitative parameters DCE (MTT) and DWI (ADCmean) of breast lesions, achieving an accuracy, sensitivity, and specificity of 94.8%, 95.3, and 94.3%, respectively. This indicates that in order to enable the most accurate breast cancer detection information on tumor cellularity, metabolism and permeability are desirable.

It is worth noting that the model based on quantitative parameters only (i.e., ADC, MTT, and SUVmax) also showed a good performance (accuracy of 93.2%).

Although the multiparametric ^18^F-FDG PET/MRI AI-based radiomics model performed best, its performance was not statistically different from the clinical interpretation by expert readers. It has to be noted, however, that while clinical interpretation achieved similar sensitivities (95.3% vs 100%), the multiparametric ^18^F-FDG PET/MRI AI-based radiomics model achieved a higher specificity (94.3% vs 73.7%), highlighting the potential of such a model to reduce false-positive findings and obviate unnecessary breast biopsies in benign breast tumors [36].

Several studies have been published on the use of AI applied to MRI for breast cancer diagnosis, mainly aiming at increasing its relatively low specificity compared to its high sensitivity, with accuracy values ranging from 72.8 to 92.0% [40–44]. Similar to our work, Zhang et al. also explored the possibility to improve the accuracy of the ML classifier combining radiomics features extracted from both morphological and functional DCE and diffusion kurtosis (DK) images of 207 histologically proven breast lesions. They found that the model based on radiomics features from T2-weighted, DKI, and quantitative DCE pharmacokinetic parameter maps had the best discriminatory ability for benign and malignant breast lesions (AUC of 0.921) [40]. In another study, radiomics coupled with ML analysis applied to DCE-MRI, including both radiomics features and clinical data, also proved to be accurate in the characterization of subcentimeter breast lesions in 96 high-risk BRCA mutation carriers, with a diagnostic accuracy of 81.5%, which was significantly higher than qualitative morphological assessment with BI-RADS classification (AUC of 53.4%) [44]. The usefulness of a multiparametric MRI approach was explored in a recent study by Tsarouchi et al. [45]. DCE and DW images of 85 breast lesions were analyzed for the extraction of first-order and texture features for the assessment of image heterogeneity and breast cancer diagnosis. Random forest resulted in the best performing algorithm (accuracy of 91.67%), combining both DCE-MRI and DWI parameters in a multiparametric assessment [45].

Regarding PET imaging, the role of this functional technique has been explored in breast cancer mainly for prognostic/therapeutic purposes, particularly in the early prediction of the response to neoadjuvant chemotherapy [46–48]. In a recently published study, the usefulness of radiomics and ML applied to PET/CT to differentiate breast carcinoma from lymphoma was investigated in a small number of lesions (19 breast lymphoma and 25 breast cancer lesions) [49]. Different predictive models were built using combinations of clinical data, quantitative parameters (SUV), radiomics features (first- and second-order parameters extracted from both PET and CT images), and CT images. Models based on clinical data, SUV, and PET radiomics features as well as on clinical data and CT radiomics features were those that were most accurate (AUC of 0.806 and 0.759 in the validation cohort, respectively) [49]. In an experimental study by Vogl et al. conducted on 34 breast lesions, a computer-aided segmentation and diagnosis (CAD) system was developed for automated lesion segmentation and classification (benign vs malignant) using separately acquired MRI and ^18^F-FDG PET/CT images [50]. The CAD system achieved a Dice similarity coefficient of 0.665 for lesion segmentation and AUC of 0.978 for breast cancer diagnosis. While PET and DWI features improved DCE-MRI segmentation performance, such an improvement was not observed for lesion characterization [50].

Limitations of our study have to be acknowledged. Firstly, our study is limited by the small sample size and the unbalanced distribution of benign and malignant breast lesions, with relevant implications for specificity. To overcome the limitation of the relatively small sample size, especially in regard to benign lesions, we opted to perform internal fivefold cross-validation which has been proven to be robust in such cases [51]. The unbalanced distribution of benign and malignant lesions is related to the fact that this study is conducted at a single tertiary care cancer center and to the inclusion criterion of only patients with BI-RADS 0, 4/5 lesions which provides the clinical indication for performing a breast ^18^F-FDG PET/MRI. We addressed this limitation by using a well-established adaptive synthetic sampling to balance the two classes. Another limitation is the lack of external validation of the proposed AI model, which may limit its generalizability. To date, there is only a limited number of centers worldwide that have clinical simultaneous PET/MRI scanners for breast imaging. Collaboration with a different institution to validate our models is in development. Furthermore, two dynamic sequences were acquired before and after an update to the clinical MRI protocol. However, acquisition parameters were similar before and after the update, and AI techniques are meant to be applied to images acquired with different acquisition protocols; indeed, this issue did not affect the level of accuracy of the ML classifier. Finally, several cases had to be excluded from the analysis as at least one among DCE-MRI, DWI, or PET images was not suitable for the extraction of quantitative parameters or for radiomics analysis, in order not to impair the reliability of our data. Despite this stringent exclusion criterion, and also considering the limited access to such an advanced imaging technique, an adequate number of breast lesions was included in the final study sample which allowed the achievement of a good performance in the AI discrimination task.

In conclusion, a simultaneous multiparametric ^18^F-FDG PET/MRI AI-based radiomics model was shown to accurately discriminate between benign and malignant breast lesions. Our initial data indicate that AI-enhanced functional and metabolic breast imaging has the potential to assist human readers in correctly classifying suspicious breast lesions and therefore obviate unnecessary invasive breast procedures. Larger multi-center studies are being planned to validate the multiparametric ^18^F-FDG PET/MRI AI-based radiomics model.

## Supplementary Information

Below is the link to the electronic supplementary material.

Supplementary file1 (DOCX 132 KB)

## Data Availability

The datasets generated during and/or analyzed during the current study are available from the corresponding author on reasonable request.
