# Supplementary material for: AI-enhanced simultaneous multiparametric 18F-FDG PET/MRI for accurate breast cancer diagnosis
Source: Eur J Nucl Med Mol Imaging. 2021 Aug 10;49(2):596–608. doi: 10.1007/s00259-021-05492-z (PMC8803815; doi:10.1007/s00259-021-05492-z)
Supplement: Supplementary file 1 — Supplementary file1 (DOCX 132 KB) [file 259_2021_5492_MOESM1_ESM.docx]

**Supplementary material**

**Table S1.** Mean values of quantitative ^18^F-FDG PET/MRI parameters in both malignant and benign breast lesions with corresponding significance levels according to independent t-test analysis.

| **Parameter** | **Malignant lesions** | | **Benign lesions** | | p value |
| --- | --- | --- | --- | --- | --- |
|  | **Mean** | **St. dev** | **Mean** | **St. dev** |  |
| Size (mm) | 28.50 | 17.09 | 19.68 | 12.56 | **0.035** |
| ADCmean (mm^2^/sec) | 0.92 | 0.24 | 1.42 | 0.23 | **< 0.0001** |
| ADCmean_cbp (mm^2^/sec) | 1.95 | 0.30 | 1.99 | 0.35 | 0.580 |
| PF (ml/100 ml/min) | 67.05 | 35.72 | 56.37 | 34.72 | 0.231 |
| VD (ml/100 ml) | 75.30 | 40.92 | 89.94 | 39.40 | 0.153 |
| MTT (sec) | 77.17 | 25.06 | 118.90 | 38.44 | **< 0.0001** |
| SUVmax | 6.22 | 4.29 | 3.09 | 3.55 | **0.003** |
| SUVmean ibp | 1.01 | 0.40 | 0.99 | 0.32 | 0.810 |
| SUVmean cbp | 0.94 | 0.39 | 1.10 | 0.41 | 0.096 |

**Note:** ADCmean = apparent diffusion coefficient of breast lesions; ADCmean_cbp = ADCmean of the contralateral breast parenchyma; PF = plasma flow; VD = volume distribution; MTT = mean transit time; SUV = standardised uptake value; St. dev = standard deviation; SUVmean_ibp = standardised uptake value of the ipsilateral breast parenchyma; SUVmean_cbp =

standardised uptake value of the contralateral breast parenchyma. In bold, significant p values (≤ 0.05).

**Table S2.** Results of the intraclass correlation coefficient analysis for intra- and inter-observer reproducibility of quantitative ^18^F-FDG PET/MRI parameter measurements.

|  | **Intra-observer** | | **Inter-observer** | |
| --- | --- | --- | --- | --- |
| **Parameter** | ICC | 95% CI | ICC | 95% CI |
| Size | 0.901 | 0.829 – 0.943 | 0.893 | 0.815 – 0.939 |
| ADCmean | 0.960 | 0.924 – 0.979 | 0.890 | 0.793 – 0.940 |
| ADCmean_cbp | 0.722 | 0.545 – 0.837 | 0.562 | 0.331 – 0.730 |
| PF | 0.882 | 0.797 – 0.933 | 0.875 | 0.786 – 0.929 |
| VD | 0.880 | 0.795 – 0.931 | 0.878 | 0.791 – 0.930 |
| MTT | 0.870 | 0.779 – 0.925 | 0.863 | 0.767 – 0.921 |
| SUVmax | 0.999 | 0.998 – 0.999 | 0.995 | 0.991 – 0.997 |
| SUVmean | 0.852 | 0.748 – 0.915 | 0.832 | 0.702 – 0.906 |
| SUVmin | 0.604 | 0.376 – 0.762 | 0.625 | 0.413 – 0.774 |
| SUVmean_ibp | 0.640 | -010 – 0.860 | 0.671 | 0.067 – 0.868 |
| SUVmean_cbp | 0.754 | 0.146 – 0.907 | 0.753 | 0.162 – 0.906 |

**Note:** ICC = intraclass correlation coefficient; ADCmean = apparent diffusion coefficient of breast lesions; ADCmean_cbp = ADCmean of the contralateral breast parenchyma; PF = plasma flow; VD = volume distribution; MTT = mean transit time; SUV = standardised uptake value; SUVmean_ibp = standardised uptake value of the ipsilateral breast parenchyma; SUVmean_cbp =

standardised uptake value of the contralateral breast parenchyma.

**Table S3.** Distribution of BI-RADS descriptors, ADC and PET scores in benign and malignant breast lesions.

| **BI-RADS Descriptor** | **Malignant lesions (n = 101)** | **Benign lesions (n = 19)** |
| --- | --- | --- |
| **FGT** |  |  |
| A | 6 (6%) | 1 (5%) |
| B | 48 (47%) | 9 (47%) |
| C | 44 (44%) | 6 ( 32%) |
| D | 3 (3%) | 3 (16%) |
| **BPE**  BPE level |  |  |
| Minimal | 62 (61%) | 5 (26%) |
| Mild | 25 (25%) | 11 (58%) |
| Moderate | 8 (8%) | 2 (11%) |
| Marked | 6 (6%) | 1 (5%) |
| BPE simmetry |  |  |
| Symmetric | 61 (60%) | 13 (68%) |
| Asymmetric | 27 (27%) | 4 (21%) |
| **Lesion type** |  |  |
| Focus | 0 (0%) | 0 (0%) |
| Mass | 74 (73%) | 15 (79%) |
| Non-mass | 6 (6%) | 2 (10.5%) |
| Mass/Non-mass | 21 (21%) | 2 (10.5%) |
| **Mass** | **Malignant lesions (n = 95)** | **Benign lesions (n = 17)** |
| Shape |  |  |
| Oval | 28 (29%) | 8 (47%) |
| Round | 19 (20%) | 1 (6%) |
| Irregular | 48 (51%) | 8 (47%) |
| Margin |  |  |
| Circumscribed | 11 (12%) | 13 (76%) |
| Irregular | 59 (62%) | 4 (24%) |
| Spiculated | 25 (26%) | 0 (0%) |
| Internal characteristics |  |  |
| Homogeneous | 7 (7%) | 4 (23%) |
| Heterogeneous | 54 (57%) | 9 (53) |
| Rim enhancement | 34 (36%) | 2 (12%) |
| Dark internal septations | 0 (0%) | 2 (12%) |
| **Non-mass enhancement** | **Malignant lesions (n = 27)** | **Benign lesions (n = 4)** |
| Distribution |  |  |
| Focal | 0 (0%) | 1 (25%) |
| Linear | 5 (19%) | 0 (0%) |
| Segmental | 7 (26%) | 1 (25%) |
| Regional | 4 (14%) | 2 (50%) |
| Multiple regions | 5 (19%) | 0 (0%) |
| Diffuse | 6 (22%) | 0 (0%) |
| Internal enhancement patterns |  |  |
| Homogeneous | 3 (11%) | 0 (0%) |
| Heterogeneous | 15 (56%) | 3 (75%) |
| Clumped | 9 (33%) | 1 (25%) |
| Clustered ring | 0 (0%) | 0 (0%) |
| **ADCmean values** | **Malignant lesions (n = 101)** | **Benign lesions (n = 19)** |
| < 1.3 x10^-3^ mm^2^/s | 96 (95%) | 5 (26%) |
| > 1.3 x10^-3^ mm^2^/s | 5 (5%) | 14 (74%) |
| **^18^F-FDG uptake** | **Malignant lesions (n = 101)** | **Benign lesions (n = 19)** |
| Higher than the background activity | 101 (100%) | 14 (74%) |
| Equal/lower than the background activity | 0 (0%) | 5 ( 26%) |

**Note:** FGT = fibroglandular tissue; BPE = background parenchymal enhancement; ^18^F-FDG **=** ^18^F-fluoro-2-deoxy-d-glucose; ADCmean = apparent diffusion coefficient of breast lesions.

**Figure S1.
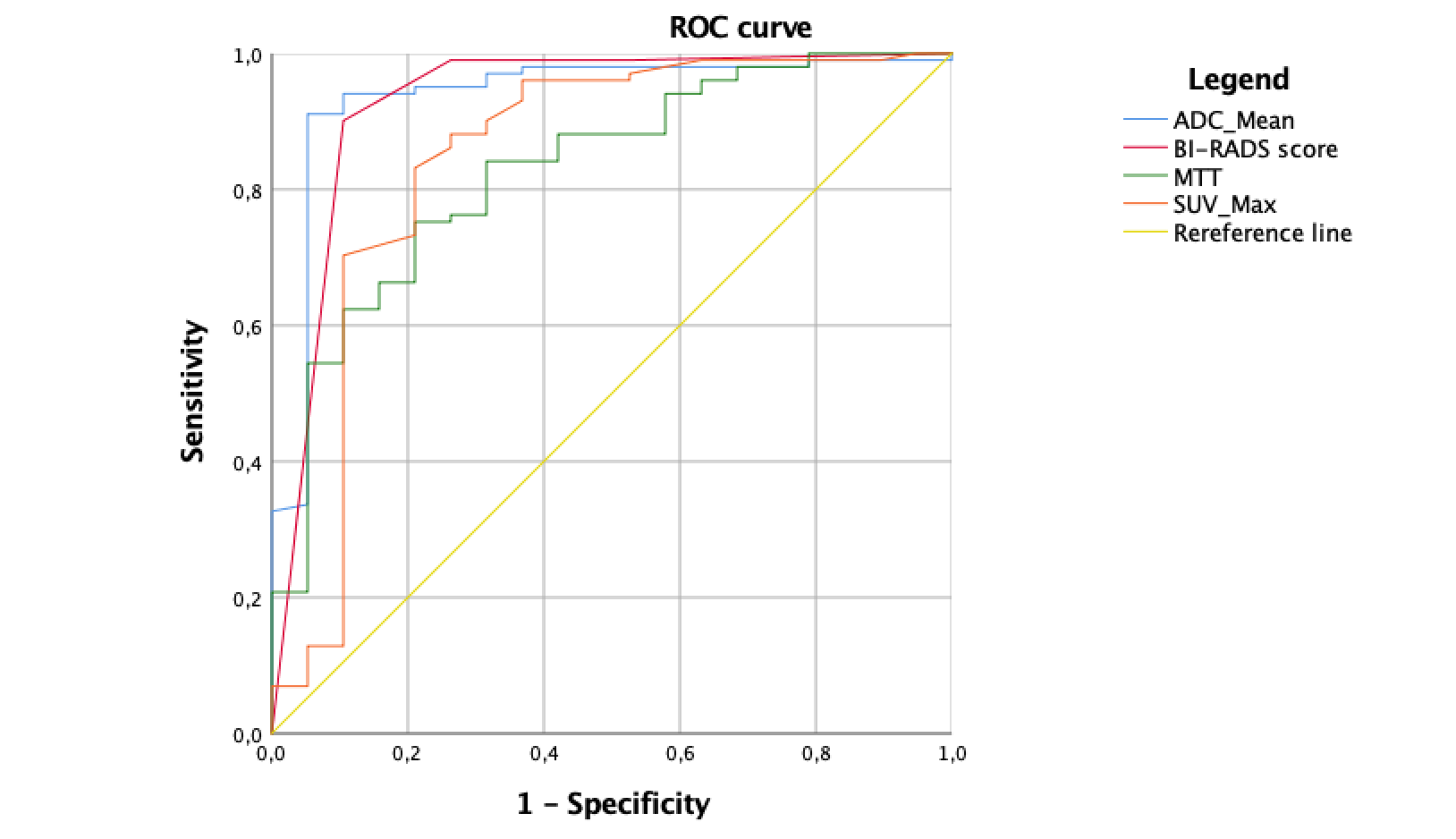
**

**Figure S4.** ROC curve analysis of BI-RADS scores (AUC = 0.929, 95% CI: 0.843-1.000) along with quantitative parameters significantly different between benign and malignant breast lesions, namely ADCmean (AUC = 0.937, 95% CI: 0.867-1.00), MTT (AUC = 0.829, 95% CI: 0.730-0.927) and SUVmax (AUC = 0.845, 95% CI: 0.721-0.970). ADCmean = apparent diffusion coefficient of breast lesions; MTT = mean transit time; SUV = standardised uptake value.

**Table S5. Accuracy metrics of BI-RADS assessment, ADC and PET alone as well as combined clinical evaluation and best radiomics model.**

|  | **Sensitivity** | **Specificity** | **PLR** | **NLR** | **AUC** |
| --- | --- | --- | --- | --- | --- |
| BI-RADS | 100 | 78.85 | 4.75 | 0.00 | 0.967 |
| ADCmean | 95.05 | 73.68 | 3.61 | 0.97 | 0.917 |
| PET | 100 | 26.32 | 1.36 | 0.00 | 0.883 |
| Combined clinical evaluation | 100 | 73.68 | 3.8 | 0.00 | 0.958 |
| Best radiomics model | 95.3 | 94.3 | 16.72 | 0.05 | 0.983 |

**Note:** ADCmean = apparent diffusion coefficient of breast lesion; PLR = positive likelihood ratio; NLR = negative likelihood ratio; AUC = area under the curve.
